# Salicylaldehyde Suppresses IgE-Mediated Activation of Mast Cells and Ameliorates Anaphylaxis in Mice

**DOI:** 10.3390/ijms23158826

**Published:** 2022-08-08

**Authors:** Tsubasa Ashikari, Masakazu Hachisu, Kazuki Nagata, Daisuke Ando, Yuki Iizuka, Naoto Ito, Kandai Ito, Yuki Ikeda, Hiroki Matsubara, Takuya Yashiro, Kazumi Kasakura, Chiharu Nishiyama

**Affiliations:** Department of Biological Science and Technology, Faculty of Advanced Engineering, Tokyo University of Science, 6-3-1 Niijuku, Katsushika-ku, Tokyo 125-8585, Japan

**Keywords:** anaphylaxis, FcεRI, IgE, mast cells, salicylaldehyde

## Abstract

Mast cells (MCs) play key roles in IgE-mediated immunoresponses, including in the protection against parasitic infections and the onset and/or symptoms of allergic diseases. IgE-mediated activation induces MCs to release mediators, including histamine and leukotriene, as an early response, and to produce cytokines as a late phase response. Attempts have been made to identify novel antiallergic compounds from natural materials such as Chinese medicines and food ingredients. We herein screened approximately 60 compounds and identified salicylaldehyde, an aromatic aldehyde isolated from plant essential oils, as an inhibitor of the IgE-mediated activation of MCs. A degranulation assay, flow cytometric analyses, and enzyme-linked immunosorbent assays revealed that salicylaldehyde inhibited the IgE-mediated degranulation and cytokine expression of bone-marrow-derived MCs (BMMCs). The salicylaldehyde treatment reduced the surface expression level of FcεRI, the high affinity receptor for IgE, on BMMCs, and suppressed the IgE-induced phosphorylation of tyrosine residues in intercellular proteins, possibly Lyn, Syk, and Fyn, in BMMCs. We also examined the effects of salicylaldehyde in vivo using passive anaphylaxis mouse models and found that salicylaldehyde administration significantly enhanced the recovery of a reduced body temperature due to systemic anaphylaxis and markedly suppressed ear swelling, footpad swelling, and vascular permeability in cutaneous anaphylaxis.

## 1. Introduction

Mast cells (MCs) and basophils are hematopoietic cells that specifically express the high affinity receptor for IgE, FcεRI, which is essential for IgE-mediated allergic responses [1]. The cross-linking of IgE bound with FcεRI on the cell surface by a multivalent antigen (Ag) induces the activation of MCs and basophils, resulting in the rapid release of various mediators, including histamine, proteases, and de novo synthesized eicosanoids as early responses, and the production of cytokines, such as TNF-α, IL-6, and IL-13, as late phase responses [2,3]. The IgE-mediated activation of MCs is an effective target for attenuating the symptoms of allergic diseases because current antiallergic drugs, including the anti-IgE humanized antibody (Ab) [4], antagonists of leukotriene receptors [5], and histamine receptors [6], inhibit events related to the IgE-mediated activation of MCs. However, these treatments are symptomatic and temporary, which are continuingly administered to patients showing allergic symptoms. Several research works have been performed to identify natural compounds that suppress the activation of MCs, which are expected to relieve allergic symptoms [7,8,9].

In the present study, we performed a screening to identify candidates for novel inhibitors of the activation of MCs by monitoring the extent of the IgE-mediated degranulation of mouse bone-marrow-derived MCs (BMMCs). As components of a library, we selected compounds that were proven to be present in and/or added as supplements to food, drink, detergents, and perfume as flavors and fragrances, because the biological safety of these compounds has been established under the conditions of their application limits; however, their activity on immunoresponses remains unknown. Through a screening, we identified salicylaldehyde, an aromatic aldehyde giving a fruity flavor, which is a natural compound contained in plant essential oils, as the most effective inhibitor of the IgE-mediated activation of MCs. In vitro experiments showed that salicylaldehyde suppressed IgE-mediated degranulation, IgE-induced cytokine release, and FcεRI expression in MCs. We investigated the effects of salicylaldehyde on IgE-mediated anaphylaxis using a mouse model and found that systemic and cutaneous anaphylactic reactions were significantly inhibited in salicylaldehyde-treated mice.

## 2. Results

### 2.1. Identification of Several Inhibitors Targeting the IgE-Mediated Activation of MCs from More Than 60 Types of Compounds

**Figure 1:**
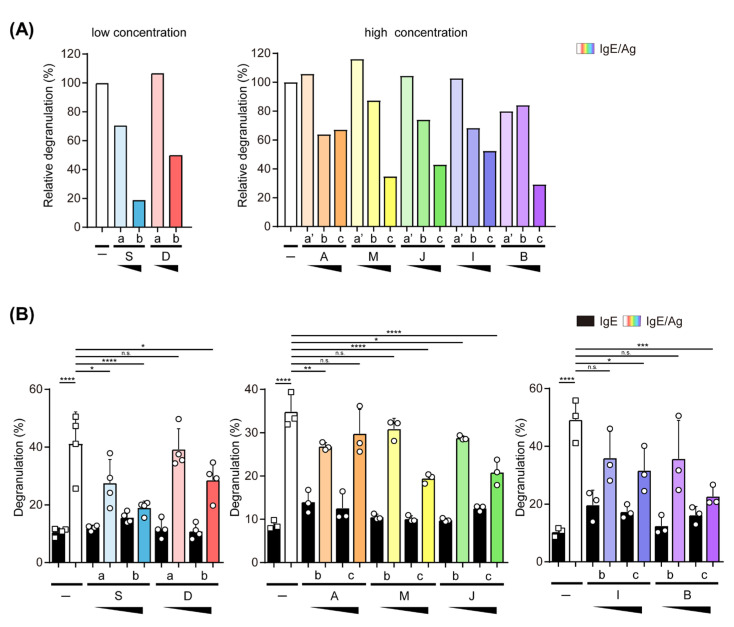
Suppression of the degranulation of MCs by candidate compounds selected from an aroma library. (**A**) Compounds passed through a screening using 0.001% *v*/*v* (left) and 0.01% *v*/*v* (right) (Table 1) were added to culture media of BMMCs at the indicated concentrations; left: a = 0.001%, b = 0.002%; right: a’ = 0.0004%, b = 0.002%, c = 0.01%; S, salicylaldehyde; D, diacetyl; A, ambrettolide; M, mintlactone; J, cis-jasmone; I, isoamyl salicylate; B, benzyl salicylate. Forty-eight hours after a preincubation with each compound, BMMCs were stimulated with anti-TNP IgE and TNP-BSA as described in the Materials and Methods Section. The relative degranulation to that of control BMMCs without compounds (=100%) is shown. (**B**) Degranulation assay data obtained from 3 independent experiments, in which different independently prepared lots of BMMCs were used; left: a = 0.001%, b = 0.002%; center and right: b = 0.002%, c = 0.01%; S, salicylaldehyde; D, diacetyl; A, ambrettolide; M, mintlactone; J, cis-jasmone; I, isoamyl salicylate; B, benzyl salicylate. Dunnett’s multiple comparison test was used for statistical analyses. *, *p* < 0.05; **, *p* < 0.01; ***, *p* < 0.001; ****, *p* < 0.0001; n.s., not significant.

To identify antiallergic compounds that inhibit the IgE-mediated activation of MCs, we conducted a screening with 63 types of compounds, as listed in Table 1. When BMMCs were stimulated with IgE and Ag after a preincubation in culture media containing 0.01% *v*/*v* of each compound for 48 h, 5 compounds were found to suppress the extent of IgE-induced degranulation by up to 60%, whereas 42 compounds did not exert apparent suppressive effects and 16 compounds decreased the viability of BMMCs. Sixteen compounds showing toxicity at 0.01% were added to the culture media of MCs under a further 1/10 diluted condition (0.001% final concentration), and two compounds were found to inhibit degranulation without affecting cell viability, eleven had no effect at 0.001%, and three were still toxic. To confirm the inhibitory effects of the candidates, we investigated the degranulation of MCs after a 48 h pretreatment with various concentrations of seven compounds (two selected at 0.001% shown in Figure 1A, left, and five selected at 0.01% shown in Figure 1A, right). In Figure 1A (left), 0.01% was not used because a concentration of 0.01% of these two compounds was judged to be toxic in Table 1. To confirm the reproducibility, we repeated the degranulation assay for three times independently, as shown in Figure 1B and obtained the result showing a significant suppression at the highest concentration of each compound except for diacetyl. Considering the concentrations (μM) calculated from the molecular weights of these compounds (Table 2), we identified salicylaldehyde as the most effective inhibitor of the IgE-induced degranulation of MCs.

### 2.2. Effects of Salicylaldehyde on the IgE-Induced Degranulation of and Cytokine Production by MCs

**Figure 2:**
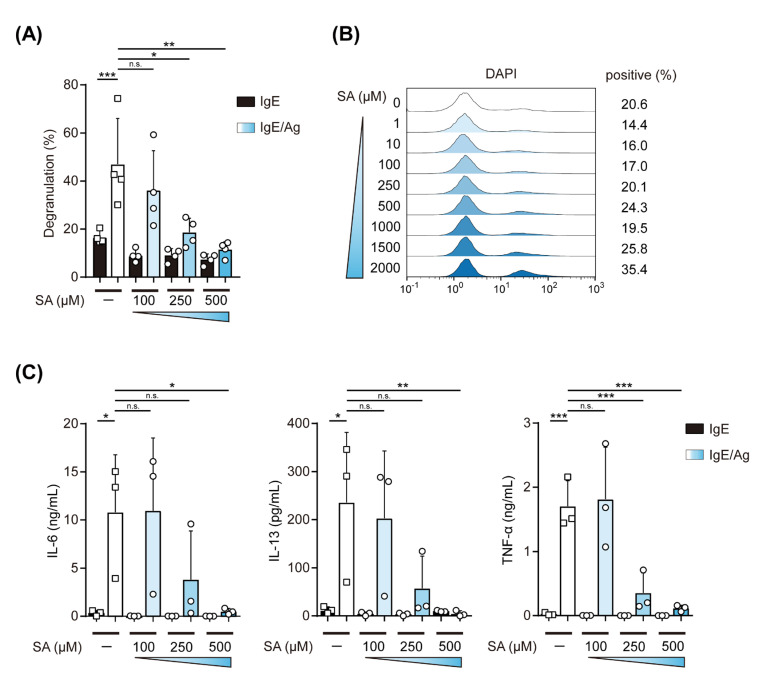
Salicylaldehyde inhibited the degranulation of and cytokine production by IgE-stimulated MCs. (**A**) Effects of salicylaldehyde on the IgE-mediated degranulation of BMMCs. BMMCs were incubated in the presence or absence of the indicated concentrations of salicylaldehyde (SA) for 48 h. After sensitization with IgE, BMMCs were incubated in Tyrode’s buffer containing TNP-BSA (IgE/Ag) or without Ag (IgE), and the supernatant was collected for β-hexosaminidase assay. (**B**) Effects of salicylaldehyde on the viability of BMMCs. BMMCs incubated with the indicated concentrations of salicylaldehyde (SA) for 48 h were treated with DAPI. DAPI-stained cells were judged to be dead cells. (**C**) Inhibition of cytokine release from BMMCs by salicylaldehyde. BMMCs incubated in the presence or absence of the indicated concentrations of salicylaldehyde (SA) for 48 h were stimulated with TNP-BSA (Ag) after sensitization with IgE. Data shown in (**A**,**C**) represent the mean ± SD of 4 and 3 independent experiments, respectively. Dunnett’s multiple comparison test (**A**,**C**) was used for statistical analyses. *, *p* < 0.05; **, *p* < 0.01; ***, *p* < 0.001; n.s., not significant.

To further evaluate the effects of salicylaldehyde on the IgE-induced activation of MCs, we measured the levels of the degranulation of IgE-stimulated BMMCs, which were treated with 100–500 μM of salicylaldehyde. As shown in Figure 2A, 250 and 500 μM of salicylaldehyde significantly suppressed IgE-mediated degranulation, while the frequency of 4′,6-diamino-2-phenylindole (DAPI)-stained cells was similar among BMMCs treated with 0–800 μM of salicylaldehyde (Figure 2B). We also examined the involvement of salicylaldehyde in the production of cytokines by IgE-stimulated BMMCs and found that the salicylaldehyde treatment inhibited the release of IL-6, IL-13, and TNF-α in a dose-dependent manner similar to that observed in the degranulation assay (Figure 2C).

These results indicate that salicylaldehyde inhibited the degranulation of and cytokine release by IgE-stimulated MCs in a dose-dependent manner.

### 2.3. Salicylaldehyde Suppressed the Expression of FcεRI and the IgE-Mediated Phosphorylation of Kinase Proteins in MC

**Figure 3:**
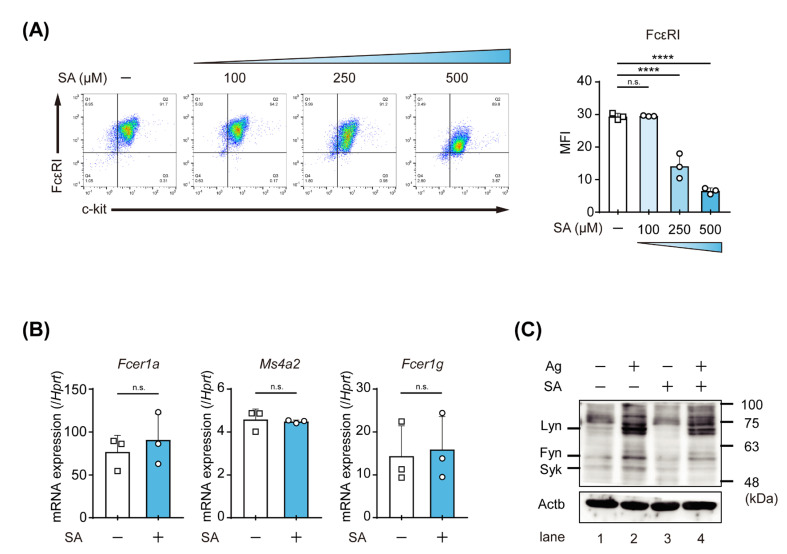
Salicylaldehyde reduced cell surface expression levels of FcεRI on MCs and IgE-mediated signal transduction in MCs. (**A**) Cell surface expression levels of FcεRI and c-kit. Typical dot plot profiles (left) and the mean fluorescence intensity (MFI) of FcεRI obtained in 3 independent experiments. BMMCs incubated in the presence or absence of the indicated concentrations of salicylaldehyde (SA) for 48 h were stained with anti-FcεRI Ab and anti-c-kit Ab. (**B**) mRNA expression levels of FcεRIα (*Fcer1a*), β (*Ms4a2*), and γ (*Fcer1g*) subunits. BMMCs pretreated with 500 μM salicylaldehyde (SA+) or its control (SA−) were harvested to assess mRNA levels by qPCR. (**C**) Western blot analysis of tyrosine-phosphorylated proteins. BMMCs treated with 500 μM salicylaldehyde for 48 h (SA+) and its control (SA−) were stimulated with IgE plus TNP-BSA (Ag) for 10 min. The expected molecular weights of Lyn, Fyn, and Syk were marked on the left. A typical result is shown, and similar results were obtained in 2 other independent experiments. Data shown in (**A** (right) and **B**) represent the mean ± SD of 3 independent experiments. Tukey’s multiple comparison test (**A** right) and the *t*-test (**B**) were used for statistical analyses. n.s., not significant; ****, *p* < 0.0001.

To identify the active sites of salicylaldehyde in the IgE-mediated activation of MCs, we examined the surface expression levels of FcεRI and c-kit on BMMCs by flow cytometry because the FcεRI expression level is associated with the degree of IgE-mediated activation of MCs [10]. Although an apparent change was not observed in BMMCs treated with 100 μM salicylaldehyde, the FcεRI expression levels were decreased by 250–500 μM salicylaldehyde in a dose-dependent manner (Figure 3A). In contrast, the c-kit expression levels on BMMCs were not reduced, even by the pretreatment with 500 μM salicylaldehyde. To clarify whether the mRNA expression levels of three subunits of FcεRI were affected by the salicylaldehyde treatment, we quantified the mRNA levels of *Fcer1a* (encoding FcεRIα), *Ms4a2* (FcεRIβ), and *Fcer1g* (FcεRIγ) in BMMCs by PCR and found that the pretreatment with 500 μM salicylaldehyde did not decrease any transcripts of the three subunits (Figure 3B). A Western blot analysis using an anti-phospho-tyrosine Ab showed that several bands, the intensities of which were apparent and/or increased in lane two (IgE-stimulated BMMCs without the salicylaldehyde treatment), were faint in lane four (IgE-stimulated BMMCs with the salicylaldehyde treatment) (Figure 3C). Based on the molecular weights of these bands, the phosphorylation of Lyn, Fyn, and Syk induced by the FcεRI-mediated stimulation appeared to be inhibited by the salicylaldehyde treatment. Considering that the level of surface FcεRI expression is closely associated with the magnitude of the FcεRI-dependent activation degree of MCs [10,11], the reduction of the IgE-induced phosphorylation of Lyn, Fyn, and Syk, which locate just downstream of FcεRIβ and γ, may reflect the decreased expression levels of FcεRI on salicylaldehyde-treated MCs.

These results indicate that salicylaldehyde suppressed the expression of FcεRI by the nontranscriptional regulation of FcεRI components and subsequently inhibited FcεRI-mediated signal transduction.

### 2.4. The Salicylaldehyde Treatment Ameliorated IgE-Mediated Anaphylaxis in Mice

**Figure 4:**
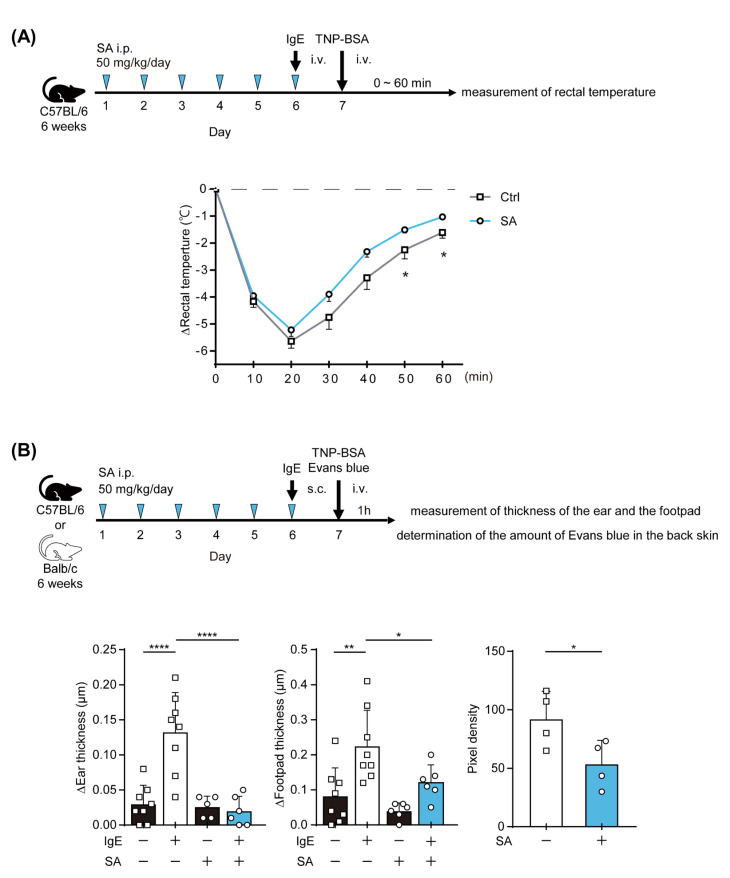
The administration of salicylaldehyde alleviated the IgE-induced anaphylactic reaction. Effects of the administration of salicylaldehyde on PSA (**A**) and PCA (**B**). Mice were intraperitonially (i.p.) administered 50 mg/kg/day salicylaldehyde (SA) or saline (Ctrl) once a day for 6 days prior to the anaphylactic reaction (**A**,**B**). (**A**) Changes in the body temperature of control and salicylaldehyde-treated mice following PSA. Open square, control (*n* = 8); open circle, administration of salicylaldehyde (*n* = 9). The *t*-test was used for statistical analyses. *, *p* < 0.05 vs. control. (**B**) Thicknesses of the ear (left panel) and footpad (center panel) of saline-treated (SA−; *n* = 8) or salicylaldehyde-treated (SA+; *n* = 6) mice 1 h after the injection with TNP-BSA. Each individual was s.c. injected with IgE (IgE+) on the right ear or right footpad and s.c. injected with saline (IgE−) on the left ear, or left footpad. The amount of Evans blue in the back skin of control (SA−; *n* = 4) or salicylaldehyde-treated (SA+; *n* = 4) Balb/c mice 1 h after the injection of TNP-BSA (right panel). Dunnett’s multiple comparison test (ear thickness and footpad thickness) and the *t*-test (Evans blue) were used. *, *p* < 0.05; **, *p* < 0.01; ****, *p* < 0.0001.

The results obtained in in vitro experiments demonstrated that salicylaldehyde inhibited the IgE-mediated activation of MCs. We utilized a passive anaphylaxis mouse model to evaluate the effects of salicylaldehyde on the function of MCs, which are activated in an IgE-dependent manner, in vivo. We tested the passive systemic anaphylaxis (PSA) model, in which a rapid decrease in body temperature was caused in mice just after an intravenous (i.v.) injection of Ag peaked 20 min after the Ag injection, and then, the body temperature was gradually recovered as observed in control (Figure 4A). By using this PSA model, we found that the administration of salicylaldehyde significantly accelerated the recovery of body temperature (open circle “SA” in Figure 4A). To further confirm the in vivo effects of salicylaldehyde, we performed passive cutaneous anaphylaxis (PCA) experiments by subcutaneously (s.c.) injecting IgE into the ear, footpad, or back skin. Ear swelling in PCA model mice 1 h after the i.v. injection of Ag was completely inhibited by the salicylaldehyde treatment (left in Figure 4B). We also confirmed that increases in footpad thickness (center in Figure 4B) and Evans blue density showing vascular permeability (right in Figure 4B) in PCA model mice were significantly suppressed in salicylaldehyde-treated mice.

Based on these results, we demonstrated that salicylaldehyde exerted beneficial effects on IgE-mediated allergic responses in vivo.

## 3. Discussion

The IgE-mediated activation of MCs plays key roles in various allergic diseases, including food allergy, anaphylaxis, and pollinosis. Previous studies examined immunomodulatory natural materials derived from plants and bacteria [12,13], and several food ingredients have been identified as inhibitors of the IgE-mediated activation of MCs [7,9,14]. In the present study, we performed a screening to identify novel antiallergic compounds from a library composed of aroma compounds, which had historically been isolated from plants and bacteria metabolites as natural compounds and sometimes chemically synthesized based on the structure of natural compounds, and whose usage conditions ensuring safety, such as application limits for each route, had been examined in detail, but whose biological activities were unknown.

In the present study, we identified salicylaldehyde as a candidate antiallergic compound through screening, in which the IgE-mediated degranulation of BMMCs was measured. We confirmed that salicylaldehyde dose-dependently inhibited the IgE-mediated degranulation and cytokine production of BMMCs. Although we suggest that a reduction in the surface expression levels of FcεRI in salicylaldehyde-treated BMMCs is involved in suppressing the IgE-mediated activation of MCs, we still consider the existence of another mechanism, because the Ca^2+^ ionophore-induced (IgE-independent) degranulation of BMMCs and LPS-induced IL-6 release by BMMCs were moderately suppressed by salicylaldehyde-treatment (Figure S1A,B). These results also suggest that other immunorelated cells and responses are regulated by salicylaldehyde. In our preliminary experiments, we found that inflammatory cytokine production by LPS-stimulated dendritic cells was inhibited by the salicylaldehyde treatment and noted that a specific inflammatory disease was ameliorated in salicylaldehyde-treated mice (data not shown). In addition, although the frequency of DAPI-stained cells was not apparently increased by the 48 h incubation in the presence of 250–500 μM salicylaldehyde, further examinations to reveal the condition of MCs are still required. We intend to perform more detailed analyses to elucidate the molecular mechanisms by which salicylaldehyde inhibits the activation of MCs and to clarify other potential applications of salicylaldehyde or its derivatives.

Salicylaldehyde is an aromatic aldehyde, *o*-hydroxyl benzaldehyde, which is industrially purified from plant essential oils and may be chemically synthesized. Although salicylaldehyde is used as a flavor and fragrance, its effects on the function of immuno-related cells remain unclear, whereas its antibacterial and antifungal activities have been investigated [15] and related compounds containing a Schiff base rather than salicylaldehyde have been reported to exhibit antitumor and antioxidant activities [16,17]. In the present study, we decided to administer 50 mg/kg salicylaldehyde to mice after preliminary experiments, which exhibited protective effects on anaphylaxis. However, it was not revealed whether this dosage amount corresponded to the concentrations of the in vitro experiments. Furthermore, although the administration of 50 mg/kg salicylaldehyde did not cause apparent acute toxicity in mice, we do not have data indicating whether the amount used in the present study is adequate or not. The kinetics of salicylaldehyde in various organs need to be examined. Salicylaldehyde was previously used as an inhibitor of the mRNA splicing of XBP1, a transcription factor, and H_2_O_2_-induced CXCL16 secretion was suppressed in salicylaldehyde-treated keratinocytes with a decrease in the production of the spliced XBP1 protein [18] and the XBP-1-dependent proliferation was also inhibited in salicylaldehyde-treated melanoma cells [19]. In contrast, in a study investigating the inhibitory activities of various salicylaldehyde-related compounds, their analogues carrying a Schiff base were found to exert inhibitory effects on XBP1 mRNA splicing [20]. In the present study, although the amount of FcεRI protein on the cell surface, which was detected with anti-FcεRIα Ab, was markedly decreased by the salicylaldehyde treatment, the mRNA levels of all three subunits were not decreased. In addition, the c-kit expression level on MCs was not affected by the salicylaldehyde treatment. These results indicate that the reduction in FcεRI on the cell surface in salicylaldehyde-treated MCs was not caused by transcriptional effects, but by other events, such as endocytosis and proteasomal degradation. The determination of intracellular protein quantities of FcεRI subunits may give us a clue to reveal the mechanisms by which salicylaldehyde reduced the cell surface’s FcεRI level. Intracellular α subunit and β subunit could be detected by the flowcytometry of permeabilized MCs [21] and the Western blotting of whole proteins of MCs [22], respectively. Nevertheless, further investigations to confirm the biological activity of salicylaldehyde and clarify the mechanisms underlying its antiallergic effects are warranted. The pathway of NRF2, which is a transcription factor that functions as a master regulator of antioxidant and antielectrophilic responses, is often identified as a target of phytochemicals [23], including resveratrol [24] and sulforaphane [25]. A recent study reported that acetyl salicylic acid (also called aspirin), a well-known nonsteroidal anti-inflammatory drug developed from salicylic acid isolated from the willow bark, activated NRF2 [26]. Therefore, we attempted to evaluate the involvement of the NRF2 pathway in the inhibitory effects of salicylaldehyde on the IgE-mediated activation of MCs. However, we have not yet been able to confirm whether the inhibition or knockout of NRF2 cancels the effects of salicylaldehyde on the activation of MCs (data not shown).

In the present study, we identified salicylaldehyde as an inhibitor of the activation of MCs by screening and demonstrated that the administration of salicylaldehyde suppressed systemic and cutaneous anaphylaxis in model mice. The identification of target molecules of salicylaldehyde and the molecular design based on the structure of target molecule(s) will contribute to the development of novel antiallergic drugs.

## 4. Materials and Methods

### 4.1. Mice and Cells

C57BL/6J and Balb/c mice were purchased from Japan SLC (Hamamatsu, Japan). All animal experiments were performed in accordance with the guidelines of the Institutional Review Board of Tokyo University of Science. The present study was approved by the Animal Care and Use Committees of Tokyo University of Science: K21004, K20005, K19006, and K18006. BMMCs were generated from mouse (C57BL/6) BM cells by cultivation under IL-3-supplemented conditions as previously reported [27].

### 4.2. IgE-Induced Degranulation of MCs

BMMCs (5 × 105) sensitized with 200 ng of anti-TNP mouse IgE (clone IgE-3, BD Biosciences, San Jose, CA, USA) in 1 mL of culture medium for 2 h were resuspended in Tyrode’s buffer containing 3 ng/mL TNP-BSA (#LSL-LG1117, Cosmo Bio, Tokyo, Japan) after washing with Tyrode’s buffer twice. The culture supernatant was harvested at 30 min after the TNP-BSA stimulation to assess β-hexosaminidase activity [28]. The extent of degranulation was shown as a ratio of the β-hexosaminidase activity to that of the whole cells, which was obtained by the lysis of MCs with Triton X-100 (Sigma-Aldrich, St. Louis, MO, USA). Salicylaldehyde (Tokyo Chemical Industry, Tokyo, Japan) was solubilized with DMSO (Sigma-Aldrich) at 500 mM or less.

### 4.3. Flow Cytometry

Flow cytometry was performed to measure the cell surface expression levels of FcεRI and c-kit with FITC antimouse FcεRIα (clone MAR-1, BioLegend, San Diego, CA, USA), and APC antimouse CD117 (clone 2B8, BioLegend), and to analyze cell viability with DAPI (#11034-56, Nacalai Tesque Inc., Kyoto, Japan). A MACS Quant Analyzer (Miltenyi Biotech, Bergisch Gladbach, Germany) and FlowJo (Tomy Digital Biology, Tokyo, Japan) were used to detect fluorescence and to analyze data, respectively.

### 4.4. ELISA

The concentrations of IL-6 and TNF-α in the culture supernatant of MCs were measured by ELISA kits purchased from BioLegend (ELISA MAX series). The mouse IL-13 DuoSet ELISA kit (DY413, R&D systems) was used to measure the concentration of IL-13. BMMCs were stimulated with anti-TNP IgE and TNP-BSA as the degranulation assay and culture supernatants were harvested at 3 h after the addition of TNP-BSA.

### 4.5. Quantification of mRNA

The preparation of total RNA from BMMCs and reverse transcription to synthesize cDNA were performed using the ReliaPrep RNA Cell Miniprep System (Promega, Madison, WI, USA) and ReverTra Ace qPCR RT Master Mix (TOYOBO, Osaka, Japan), respectively. The mRNA levels of FcεRIα, β, and γ subunits were measured by quantitative PCR using the Step-One Real-Time PCR system (Applied Biosystems, Waltham, MA, USA) with THUNDERBIRD SYBR qPCR Mix (Toyobo) and the following primers: *Fcer1a* forward; 5′-GAGTGCCACCGTTCAAGACA-3′, *Fcer1a* reverse; 5′-GTAGATCACCTTGCGGACATTC-3′, *Ms4a2* forward; 5′-TGGTTGGTTTGATATGCCTTTGT-3′, *Ms4a2* reverse; 5′-CACTGCACCCCAGAATGGATA-3′, *Fcer1g* forward; 5′-ATCTCAGCCGTGATCTTGTTCT-3′, *Fcer1g* reverse; 5′-ACCATACAAAAACAGGACAGCAT-3′, *Hprt* forward; 5′-TCCATTCCTATGACTGTA GATTTTATCAG-3′, *Hprt* reverse; 5′-AACTTTTATGTCCCC CGTTGACT-3′.

### 4.6. Western Blot Analysis

BMMCs (1.0 × 10^6^) were collected 10 min after the stimulation with anti-TNP IgE and TNP-BSA. Subsequent steps, including the electrophoresis of cell lysates and transfer of proteins from acrylamide gels to membranes, were performed as previously described [29] using anti-phospho-tyrosine (clone 4G10, Sigma-Aldrich) and anti-β-actin (clone AC-15, Sigma-Aldrich) Abs.

### 4.7. PSA and PCA

To induce PSA, 6-week-old C57BL/6 female mice, which were i.v. injected with 3 μg of anti-TNP IgE, were i.v. injected with 20 μg of TNP-BSA at 24 h after the IgE injection. The body temperature of mice was measured by a weighing environment logger (AD-1687, A&D) every 10 min after the TNP-BSA injection for 1 h.

To establish PCA, 20 μg of TNP-BSA and 0.5% Evans blue (Fujifilm Wako Chemicals, Osaka, Japan) were i.v. injected into mice s.c. preinjected with anti-TNP IgE or saline as a control into the ear (1.0 μg IgE, C57BL/6 female), footpad (0.02 μg IgE, C57BL/6 female), or back (0.5 μg IgE, Balb/c female). The thicknesses of the ear and footpad were measured at 1 h after the TNP-injection and back skin was collected to calculate blue staining caused by permeabilized Evans blue using a densitometric analysis with Image J (NIH, Bethesda, MD, USA) as previously reported [30].

### 4.8. Statistical Analysis

The two-tailed Student’s t-test (to compare two samples) and a one-way ANOVA-followed by Tukey’s multiple comparison test or Dunnett’s multiple test (for more than three samples) was used, and *p* values <0.05 were considered to be significant.

## Figures and Tables

**Table 1:** List of compounds subjected to screening.

No.	Compound	0.01%	0.001%	No.	Compound	0.01%	0.001%
1	Furfural	−		33	Anisole	−	
2	2-Piperonylpropanal	X	−	34	Benzaldehyde propylene glycol acetal	−	
3	Salicylaldehyde	X	+	35	Β-Caryophyllene	X	−
4	Phenylmethanol (benzylalchol)	−		36	*p*-Cymene	−	
5	Furfuryl alcohol	X	−	37	Diacetyl	X	+
6	2-Acetyl-3-octanone	−		38	1.8-Cineole	−	
7	Triacetin	−		39	Acetic acid	−	
8	Isobutyl acetate	−		40	Hexanoic acid	−	
9	Butyl butyryllactate	−		41	Hydroxycitronellal diethyl acetal	−	
10	Diethyl succinate	−		42	Levulinic acid	−	
11	δ-Dodecalactone	−		43	α-Pinene	X	−
12	Ethyl acetoacetate	−		44	Terpinolene	−	
13	Ethyl dodecanoate	−		45	2-Pentylfuran	−	
14	Ethyl nonanoate	−		46	Bisabolene	X	−
15	Ambrettolide	+		47	Hexanal propylene glycol acetal	−	
16	Methyl phenylacetate	−		48	1.4-Cineole	−	
17	Triethyl citrate	−		49	Isovaleraldehyde diethyl acetal	−	
18	γ-Pentlactone	−		50	Carvacrol	X	−
19	Methyl *epi*-dihydrojasmonate	−		51	4-Ethylguaiacol	X	−
20	Hexyl phenylacetate	−		52	Nerolidol	X	−
21	Ethylene brassylate	−		53	Hotrienol	−	
22	*Cis*-3-Hexenyllactate	−		54	α-Hexylcinnamaldehyde	X	−
23	*Cis*-3-Hexenylpyruvate	−		55	*trans*-2-Heptenal	X	X
24	Methyl 3-hydroxybutyrate	X	−	56	5-Methyl-2-phenyl-2-hexenal	X	X
25	Isoamyl salicylate	+		57	2-Phenylcrotonaldehyde	X	−
26	Benzyl salicylate	+		58	2-Isopropyl-5-methyl-2-hexenal	−	
27	2-Hydroxy-3-methyl-2-hexen-1,4-olide	−		59	Linalool-3.6-oxide	−	
28	Allyl cinnamate	−		60	α-Irone	X	X
29	Allyl phenoxyacetate	−		61	*cis*-Jasmone	+	
30	Furaneol acetate	−		62	4-Oxoisophorone	−	
31	Mintlactone	+		63	Mesifurane	−	
32	Isoamyl alcohol	−					

+: inhibition; − no effect; X: toxic; blank: not tested.

**Table 2:** Molecular weights and concentrations of compounds analyzed in Figure 1.

Compound	MW	v/v (%)	Concentration (μM)
Salicylaldehyde	122.1	0.001	95.8
		0.002	191.6
Diacetyl	86.1	0.001	115.0
		0.002	230.0
Compound	MW	v/v (%)	Concentration (μM)
Ambrettolide	252.4	0.002	75.8
		0.010	378.8
Isoamyl salicylate	208.3	0.002	100.8
		0.010	504.2
Benzyl salicylate	228.2	0.002	102.5
		0.010	512.6
Mintlactone	166.2	0.002	127.3
		0.010	636.5
*cis*-Jasmone	164.2	0.002	114.5
		0.010	572.3

## Data Availability

Not applicable.

## Supplementary Materials

The following supporting information can be downloaded at: https://www.mdpi.com/article/10.3390/ijms23158826/s1.
