# Supplementary material for: Salicylaldehyde Suppresses IgE-Mediated Activation of Mast Cells and Ameliorates Anaphylaxis in Mice
Source: Int J Mol Sci. 2022 Aug 8;23(15):8826. doi: 10.3390/ijms23158826 (PMC9368859; doi:10.3390/ijms23158826)
Supplement: Supplementary file 1 [file ijms-23-08826-s001.zip › ijms-1821970-supplementary.pdf]

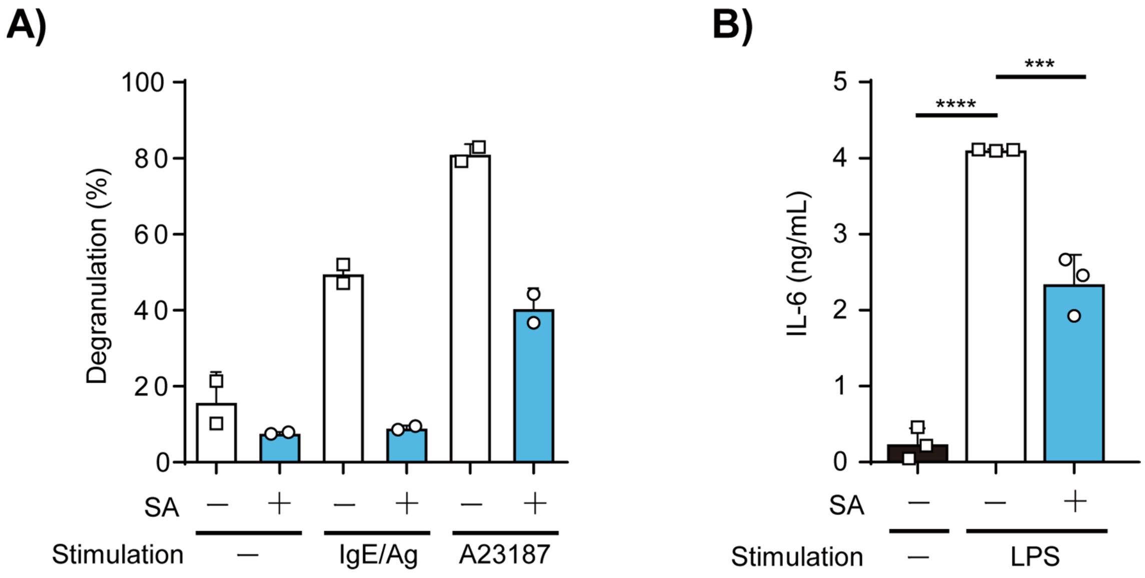


Figure S1. The effects of salicylaldehyde on IgE-independent stimulation of MCs.

(**A**) Degranulation degree of BMMCs. BMMCs incubated in the presence (SA +) or absence (SA -) of salicylaldehyde for 48 h were stimulated with $Ca^{2+}$ ionophore (A23187; 1 μM). n = 2.

(**B**) The effect of salicylaldehyde on LPS-induced IL-6 production by BMMCs. BMMCs treated with salicylaldehyde (SA +) and its control (SA -) were stimulated with 1 μg/mL LPS for 3 h. n = 3. Dunnett's multiple comparison test was used. ***, $p<0.001$; ****, $p<0.0001$.
